# Microvessel Density and Clinicopathologic Characteristics in Hepatocellular Carcinoma With and Without Cirrhosis

**Published:** 2007-02-14

**Authors:** Ivan Chebib, Meer Taher Shabani-Rad, Michelle S. Chow, James Zhang, Zu-hua Gao

**Affiliations:** Department of Pathology and Laboratory Medicine, University of Calgary and Calgary Laboratory Services, Calgary, Alberta, Canada

**Keywords:** hepatocellular carcinoma, cirrhosis, microvessel density, tissue microarray, immunohistochemistry

## Abstract

Angiogenesis is essential to the survival, growth, invasion, and metastasis of various human solid tumors. We compared the microvessel density (MVD) and clinicopathologic features of two different groups of hepatocellular carcinoma (HCC), namely HCC with cirrhosis (HCC-C) and without cirrhosis (HCC-NC). A tissue microarray composed of 20 normal livers, 20 cirrhotic livers, tumor and adjacent background non-neoplastic liver tissues from 20 HCC-C and 20 HCC-NC were constructed and stained immunohistochemically with antibodies against the antigen CD34. The MVD was determined by the measurement of the area and density of CD34 positive sinusoidal endothelial cells using the Image Pro Plus software. There was a trend of increased MVD in cirrhotic liver compared to normal liver and in cirrhotic background non-neoplastic liver adjacent to the tumor compared to the non-cirrhotic background non-neoplastic liver. Tumor tissue of HCC-C and HCC-NC both showed significantly higher MVD than their adjacent background non-neoplastic liver tissue. There was no statistical difference in MVD between HCC-C and HCC-NC. A higher value of MVD was seen in tumors of intermediate size (5–10 cm), high histologic grade, the presence of lymphvascular space invasion, and the underlying etiology of hepatitis C and alcoholic steatohepatitis. This data indicates that MVD may play an important role in liver carcinogenesis and neoplastic progression. The difference in clinical behavior between HCC-C and HCC-NC does not seem to be associated with differences in tumor MVD. Objective measurement of MVD using standardized computer software could potentially be used as a clinical marker to predict patients’ prognosis.

## Introduction

Hepatocellular carcinoma (HCC) is the sixth most common cancer worldwide in terms of numbers of cases but because of its very poor prognosis, it is the third most common cause of death from cancer (Parkin et al. 2005). HCC is multifactorial in etiology and complex in pathogenesis. The major risk factor is liver cirrhosis associated with chronic hepatitis B and hepatitis C infection, alcohol, aflatoxin B exposure, and various metabolic disorders (Harris, 1990; Saito et al. 1990; Chen et al. 1996; Johnson, 1987). However, in approximately 10–40% of patients the tumor arises from a non-cirrhotic liver (Nazeako et al. 1996). Patients with HCC with cirrhosis (HCC-C) are significantly older. The tumor from patients with cirrhosis tends to be less well differentiated (high grade), exhibits local portal invasion and metastasis more often than tumors from patients without cirrhosis. Survival advantages of HCC without cirrhosis (HCC-NC) over those with cirrhosis have been well documented (Nazeako et al. 1996; Kew, 1989; Okuda et al. 1989). However, the underlying mechanism for these clinicopathologic differences is yet uncertain.

The sinusoids of liver are lined by a unique endothelium structurally characterized by the presence of fenestrations forming the so-called sieve plates and the absence of a subenthothelial basement membrane (Philips et al. 1987). Phenotypically, the sinusoid endothelial cells are characterized by the expression of specific markers such as CD4, CD14 and CD32, and by the lack of expression of normal capillary endothelial markers such as CD31 and CD34 (Scoazec et al. 1991; Frachon et al. 2001). In hepatocellular carcinoma, the sinusoid endothelial cells lost their structural and phenotypic characteristics and adopted the structure and phenotype of normal capillary endothelial cells, a phenomenon known as capillarization (Kin et al. 1994; Nakamura et al. 1997). The association of increased expression of vascular endothelial growth factor, basic fibroblast growth factor and hypoxia-inducible factor 1-alpha with the capillarization of sinusoidal endothelial cells demonstrated in recent studies indicate that the ‘capillarization’ phenomenon is not merely a change of endothelial cell differentiation, but rather represents a process of tumor angiogenesis (Park et al. 2000; Imura et al. 2004; Huang et al. 2005). Tumor angiogenesis is critical for providing nutrient supply to the tumor and providing the route for tumor metastasis. Microvessel density (MVD), a quantitive measurement of tumor angiogenesis has been shown to be of prognostic value in many types of malignancy including HCC (Weidner, 1995; Poon et al. 2002).

Variable degrees of sinusoidal endothelial capillarization have also been observed in the cirrhotic liver of human and animal models (Huet et al. 1985; Lu X et al. 2003; Xu et al. 2003), which became more consistent in dysplastic nodules in cirrhotic liver. Therefore, capillarization of sinusoidal endothelial cells was considered part of the carcinogenesis process in HCC-C. HCC-NC lacks the process of pre-existing hepatitis and cirrhosis. There is no study yet to address the differences of MVD in the tumor and its adjacent background non-neoplastic liver between HCC and HCC-NC. In this study, we used the area and intensity of CD34 positive sinusoid endothelial cells as a measurement of MVD and compared their value between normal liver and cirrhotic liver, between the non-neoplastic background liver adjacent to HCC-C (HCC-CB) and the non-neoplastic background liver adjacent to HCC-NC (HCC-NCB), and between the tumor tissue of HCC-C and HCC-NC. The quantitive data of MVD was then analyzed in correlation with the clinicopathologic characteristics.

## Materials and Methods

### Tissue samples

With the approval of the institutional review board of the University of Calgary, we retrieved the following tissue samples from the Department of Pathology, University of Calgary: 20 normal livers (15 men and 5 women; age range 29–100 years), 20 cirrhotic livers (11 men and 9 women; age range 39–89 years), tumors and their adjacent background non-neoplastic livers from 20 HCC-C (15 men and 5 women, age range 44–75 years) and 20 HCC-NC (17 men and 3 women, age range 25–85 years). The tissue samples of normal liver and cirrhotic liver were obtained from well-preserved autopsy specimens. The tissue samples of tumors and their adjacent background non-neoplastic livers were obtained from surgical resection specimens. Routine hematoxylin-eosin stained tissue sections were reviewed by 2 pathologists (IC, ZG) and the tumors were graded using the WHO grading system (Hirohashi, 2000). HCC-NC was defined by the absence of bridging fibrosis in the background non-neoplastic liver tissue. Representative areas were selected for the construction of the tissue microarray blocks using 1.0 mm punchers on the manual tissue arrayer MTA-1 (Beecher Instruments, Sun Prairie, WI). The background non-neoplastic liver tissue was sampled within 0.5 cm distance to the tumor. The tumor tissue was sampled from growing edge where the maximal MVD is believed to exist (Weidner, 1995).

### Clinical information

The demographic data, background liver diseases, date of HCC diagnosis, date and vital status (living or deceased) of last follow up, cause of death (due to HCC or other causes), disease-specific survival (from date of diagnosis to date of death), tumor size, location, stage, type of surgery, recurrences, intra- and extrahepatic metastasis were obtained by reviewing the patients’ medical charts and communication with attending physicians. Patients were censored at the latest date when they were seen alive or at the date of their non-tumor-related death.

### Immunohistochemistry

Immunohistochemical staining was performed on 4 μm sections obtained from formalin-fixed, paraffin-embedded tissue microarray blocks. After deparaffinization and rehydration, tissue sections were incubated with monoclonal antibodies against CD34 (mouse clone QBEnd/10, dilution 1:200, Novocastra, Norwell, MA). A subsequent reaction was performed with biotin-free horseradish peroxidase labeled polymer of EnVision plus detection system (DakoCytomation, Carpinteria, CA). A positive reaction was visualized with diaminobenzidine solution followed by counterstaining with hematoxylin. Placenta tissue was used as the positive control according to the manufacturers’ recommendation. Negative controls were prepared by using non-immune mouse IgG.

### Evaluation of MVD

The evaluation of MVD was performed without knowledge of clinicopathologic data. The intensity and area of sinusoidal endothelial staining were quantitively measured using the Image Pro Plus 5.0 software (Media Cybernetics, Inc, Silver Spring, MD). The entire tissue core (1 mm^2^) of each case in the array excluding the microvessel and connective tissue was measured at a magnification of 100x. The images were then imported into the Image-Pro Plus software, where they were calibrated to a known area of measurement. The immunohistochemical stain was then selected using the “color selection” function and the “area/density (intensity) measurement” functions were used to calculate the respective values. These values were then transferred onto the clipboard and placed into a Microsoft Excel worksheet.

### Statistical Analysis

The data were compiled in a Microsoft Excel spreadsheet and analyzed using STATA 9.0 software (StataCorp, College Station, TX). Fisher’s exact test and chi-square analysis were used for comparing the clinicopathologic parameters between HCC-C and HCC-NC. Survival data were analyzed by Kaplan-Meier survival estimates and log-rank tests. Non-parametric multiple analysis of variance (ANOVA) was used to see the effect of a categorical variable (normal liver, cirrhotic liver, etiology, histologic grade, tumor size, etc) on dependent variables (CD34 area and intensity). Student t-test was used to compare paired samples i.e. HCC-C vs. HCC-CB and HCC-NC vs. HCC-NCB. Mann-Whitney U test was used for the comparison of unpaired samples, i.e. HCC with and without lymphvascular invasion.

## Results

### Clinical and pathologic characteristics

**Figure 1:**
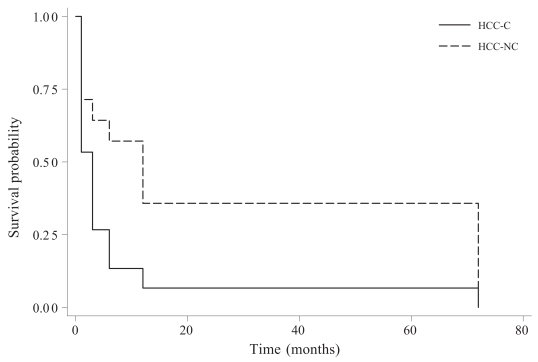
Kaplan-Meier survival estimates. In comparison with hepatocellular carcinoma patients with cirrhosis. (HCC-C), hepatocellular carcinoma patients without cirrhosis (HCC-NC) showed a significantly longer disease-free survival (p < 0.05, log-rank test).

Comparison of clinicopathologic parameters between hepatocellular carcinomas with and without cirrhosis is summarized in Table 1. Hepatitis of virus and alcoholic etiology were more commonly seen in patients suffering from HCC-C. In the HCC-NC group, less than 1/2 were normal, an equal number had diagnosable disease, and 4 had “unknown” hepatitis. Despite the lack of statistical differences between HCC-C and HCC-NC in patients’ age, gender, tumor histologic type (except fibrolamellar carcinoma only seen in HCC-NC), grade, size, multiplicity, lymphvascular space invasion, tumor stage and surgical margins, patients with HCC-NC enjoyed a significantly longer period of disease-free survival than patients with HCC-C (Figure 1).

### The area and intensity of sinusoidal CD34 positive cells

**Figure 2:**
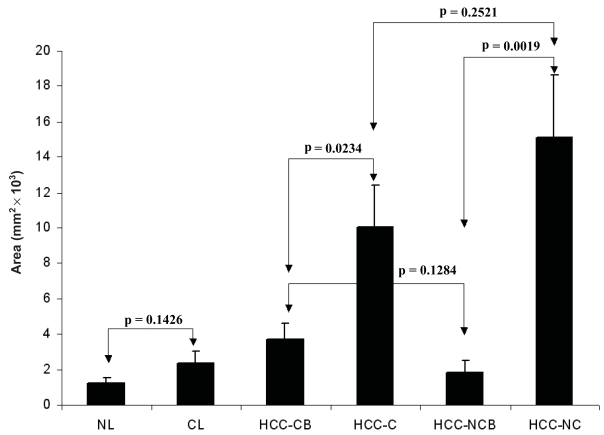
Area of CD34 positive sinusoidal endothelial cells in different types of tissue. CL: cirrhotic liver; HCC-CB: non-neoplastic cirrhotic background liver tissue adjacent to the tumor; HCC-C: tumor tissue of HCC with cirrhosis; HCC-NCB: non-neoplastic non-cirrhotic background liver tissue adjacent to the tumor; HCC-NC: tumor tissue of HCC without cirrhosis; NL: normal liver.

**Figure 3:**
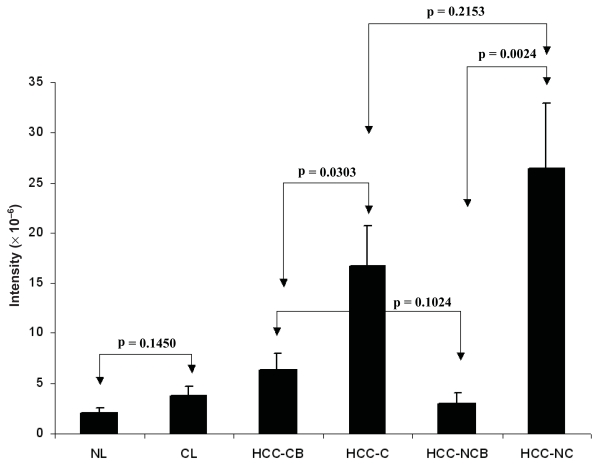
Intensity of CD34 positive sinusoidal endothelial cells in different types of tissue. CL: cirrhotic liver; HCC-CB: non-neoplastic cirrhotic background liver tissue adjacent to the tumor; HCC-C: tumor tissue of HCC with cirrhosis; HCC-NCB: non-neoplastic non-cirrhotic background liver tissue adjacent to the tumor; HCC-NC: tumor tissue of HCC without cirrhosis; NL: normal liver.

**Figure 4:**
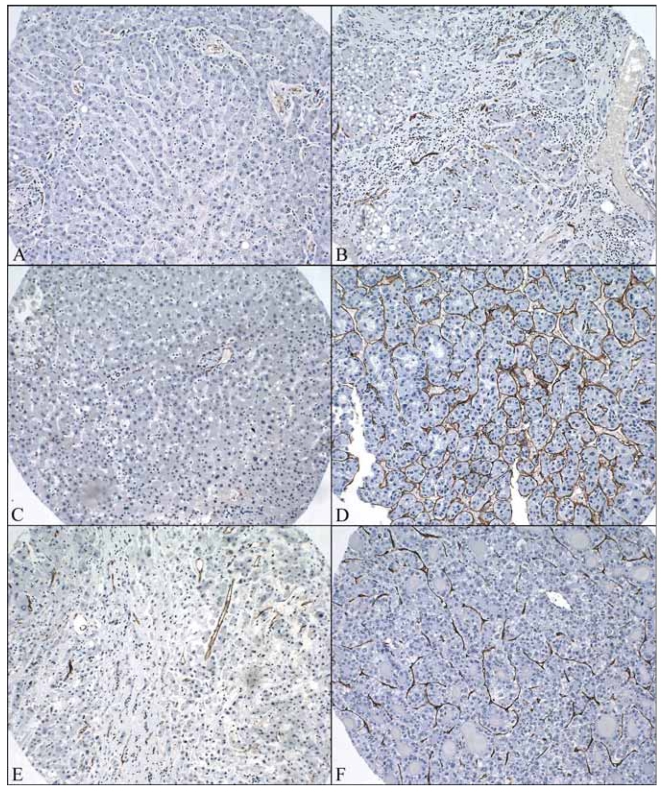
Representative images of CD34 staining in different types of tissue (original magnification x200). **A**) CD34 staining in normal liver. **B**) CD34 staining in cirrhotic liver without tumor. **C**) CD34 staining in the background non-neoplastic liver tissue adjacent to HCC without cirrhosis. **D**) CD34 staining in the tumor tissue of HCC without cirrhosis. **E**) CD34 staining in the background non-neoplastic liver tissue adjacent to HCC with cirrhosis. **F**) CD34 staining in the tumor tissue of HCC with cirrhosis.

Figure 2 shows the area of CD34 positive sinusoidal endothelial cells in different types of tissue. Figure 3 shows the intensity of CD34 positive sinusoidal endothelial cell staining in different types of tissue. Figure 4 shows the representative images of CD34 staining in different types of tissue. There was a trend of increased CD34 positive area and intensity in cirrhotic livers than normal livers and in HCC-CB than HCC-NCB. There was no significant difference between HCC-C and HCC-NC in both area and intensity of CD34 sinusoidal endothelial cell staining. Comparisons with their relevant background non-neoplastic liver tissue, both HCC-C and HCC-NC showed a statistically significant increase in both area and intensity of CD34 sinusoidal endothelial cell staining.

### Correlation of microvessel intensity with clinicopathologic characteristics

Table 2 shows the correlation of the mean value of both the area and intensity of CD34 sinusoidal endothelial staining with the presence or absence of lymphvascular space invasion, tumor size, histologic grade and the underlying etiology. Although not statistically significant, a higher MVD value was seen in HCCs with lymphvascular space invasion, high histologic grade, medium tumor size (5–10 cm), and the underlying etiology of hepatitis C and alcoholic steatohepatitis.

## Discussion

Von Willebrand’s factor (vWF), CD31, and CD34 are commonly used markers for highlighting endothelial cells in normal tissue. Antibodies against vWF and CD31 have failed to stain sinusoid endothelial cells in many HCC cases, whereas CD34 has proven to be a more sensitive and specific endothelial cell marker for microvessels in HCC (McComb et al. 1982; Simmons et al. 1990; Ruck et al. 1995). The presence of CD34 positive, vWF-negative sinusoidal endothelial cells in HCC was further verified by more detailed comparative studies (Tanigawa et al. 1997; Ohmori, 2001). CD34 is a 110-kd, heavily glycosylated transmembrane protein that is present in hematopoietic progenitor cells and endothelial cells (Fina et al. 1990). Due to its preferential expression on the surface of regenerating or migrating endothelial cells, CD34 has been used as a marker of proliferating endothelial cells in the growing sprouts during angiogenesis (Schlingermann et al. 1990). It has been recently demonstrated that bone-marrow-derived CD34 positive endothelial progenitor cells migrate through the blood circulation and are incorporated into the site of angiogenesis (Asahara et al. 1997; Shi et al. 1998). In this study, we attempted to stain our tissue array with antibodies against CD31 (data not shown), vWF (data not shown) and CD34. Only CD34 showed consistent positive staining in tumor tissue. Our data is therefore analyzed based on the intensity and area of CD34 positive sinusoidal endothelial cell staining in different types of tissue. Manual microscopic count was used previously for the assessment of intratumor microvessel density (Mazzanti et al. 1997; Tanigawa et al. 1997; Sun et al. 1999; Poon et al. 2002; Lu et al. 2004). A computer image analyzer was used in this study for the assessment of the area and intensity of immunohistochemical staining to increase accuracy and to avoid inter-observer variation. The standardized technology provides more objective measurements of MVD and makes it more feasible for future clinical applications.

We found a trend of higher MVD in cirrhotic liver than normal liver and in HCC-CB than HCC-NCB. This observation is consistent with reports from other authors (El-Assal et al. 1998;Yamamoto et al. 1998; Ruck et al. 1995; Cui et al. 1996; Frachon et al. 2001). The increased MVD in cirrhotic liver was generally considered part of carcinogenesis process in HCC arising from cirrhotic liver (Messerini L. et al. 2004). Despite the lack of pre-existing cirrhosis, HCC-NC also had a significantly higher MVD than the background non-neoplastic liver. Therefore, increased MVD is not unique for HCC-C, but rather a universal process associated with neoplastic transformation in HCC. The significant differences in MVD between tumor tissue and their background non-neoplastic liver tissue in both HCC-C and HCC-NC suggests the association of sinusoidal capillarization with neoplastic transformation irrespective of the pre-existence or absence of cirrhosis. The clinical difference between patients with HCC-C and patients with HCC-NC seems unrelated to microvessel density in the tumor and their surrounding non-neoplastic liver tissue.

Reports about the association of underlying liver diseases with the MVD in the HCC and non-neoplastic liver have been conflicting (El-Assai et al. 1998; Mazzanti et al. 1997; Ohmori et al. 2001; Urashima et al. 1993). The most recent study suggests that HCV associated HCC has a significantly higher value of MVD both in the tumor and non-tumorous areas compared to the equivalent in HBV, which indicate a closer link between HCV infection with angiogenesis and hepatocarcinogenesis (Messerini et al. 2004). In our group, HCV-HCC had a higher MVD than HBV-HCC. More interestingly, we found that alcohol-associated HCC had the highest value of MVD. Although the association between early alcoholic liver diseases with capillarization of sinusoids has been documented in previous studies (Popper et al. 1981; Horn et al. 1985; Urashima et al. 1993), our study, for the first time, demonstrated a high value of intratumoral MVD in the alcoholic steatohepatitis related HCC. In our study group, a significant number of patients with HCC-NC (25%) had underlying alcoholic steatohepatitis, which might contribute to the increased MVD in HCC-NC. The differences in MVD among HCC arising from different liver diseases are an interesting area deserving further investigation.

Angiogenesis is critical for tumor growth because it provides the oxygen and nutrition that is required by the proliferating malignant cells. Unlike other solid tumors, increased MVD was only observed in small to medium sized HCCs. Some have reported an inverse correlation between tumor size and MVD in HCC (Ng et al. 2001; Poon et al. 2002; Lu et al. 2004). Based on their study of 71 resected hepatocellular carcinomas, El-Assal et al. (1998) concluded that angiogenesis plays a fundamental role in the tumor proliferation of HCCs 2–5 cm in diameter, whereas, the importance of neovascularization is reduced as the tumors became larger. Similarly, Tanigawa et al. (1997) found increased vascularity in tumors greater than 2 cm. We found that tumors of 5–10 cm in diameter had the highest MVD compared with larger and smaller sized tumors. Tumors that fall into this size range may be in an ideal condition for receiving nutritional supply from the circulation. Larger sized tumors with lower MVD may have perfusion deficiencies, which will eventually lead to tumor necrosis. On the other hand, tumors of 5 to 10 cm in diameter may be at the best size window for chemotherapy due to the relative easiness in transporting medications into the tumor.

The association of high MVD-CD34 with high histological grade, the presence of lymphvascular space invasion, the presence of satellite lesions, high post-surgical tumor recurrence rate, high metastasis rate and poor disease-free survival has been observed in many studies (Tanigawa et al. 1997; El-Assal et al. 1998; Sun et al. 1999; Poon et al. 2002; Zhang et al. 2006; Yamamoto et al. 1998; Yao et al. 2005). The trend of increased MVD with the progression of HCC from low to high histologic grade and the presence of lymphvascular space invasion observed in this study further suggests the association of MVD with the progression of HCC. Therefore, it would be conceivable to speculate that a higher level of MVD might be an indicator of poor prognosis.

In summary, tumor tissue of HCC-C and HCC-NC both showed significantly higher microvessel density than their adjacent background non-neoplastic tissue. There was no statistical difference in MVD between HCC-C and HCC-NC. A higher level of MVD was seen in tumors of intermediate size (5–10 cm), high histologic grade, the presence of lymphvascular space invasion, and the underlying etiology of hepatitis C and alcoholic steatohepatitis. This data indicates that MVD may play an important role in liver carcinogenesis and neoplastic progression irrespective of the pre-existence or absence of cirrhosis. The difference in clinical behavior between HCC-C and HCC-NC does not seem to be associated with differences in tumor MVD. Objective measurement of MVD using standardized computer software could potentially be used as a clinical marker to predict patients’ prognosis.

## Figures and Tables

**Table 1:** Comparison of clinicopathologic parameters between hepatocellular carcinoma with and without cirrhosis.

Parameters	HCC-C	HCC-NC	p value*
Age			0.707
< 50 yrs	6	4	
>50 yrs	14	16	
Gender			0.695
Male	15	17	
Female	5	3	
Background liver disease			0.002
Hepatitis B	8	2	
Hepatitis C	7	1	
Non-specific hepatitis	0	4	
Alcoholic steatohepatitis	3	5	
None	2	8	
Histology type			0.231
Classic	20	17	
Fibrolamellar	0	2	
Sclerosing	0	1	
Tumor size			0.200
<5 cm	11	6	
>5 cm	9	14	
Tumor multiplicity			1.000
Solitary	14	13	
Multiple	6	7	
Tumor Grade (WHO)			0.381
I	0	2	
II	17	14	
III	3	4	
IV	0	0	
Lymphvascular invasion			0.523
Present	7	10	
Absent	13	10	
Tumor Stage			0.875
I	10	10	
II	4	3	
III	3	5	
IV	3	2	
Surgical margin	all negative	all negative	
	3 N/A	4N/A	

HCC-C: hepatocellular carcinoma with cirrhosis; HCC-NC: hepatocellular carcinoma without cirrhosis.

* Fisher’s exact test and chi-square analysis.

**Table 2:** Correlation of microvessel density with clinicopathologic characteristics.

	CD34 area (mean, mm^2^)	CD34 intensity (mean × 10^5^)	p value Statistical analysis
Lymphvascular invasion			Area: 0.927
Yes	0.015	249	Intensity: 0.783
No	0.011	194	Mann-Whitney test
Histologic grade			
I	0.000	1.3	Area: 0.164
II	0.011	192	Intensity: 0.173
III	0.020	360	ANOVA test
Tumor size			
<5 cm	0.008	154	Area: 0.276
5–10cm	0.018	314	Intensity: 0.329
>10 cm	0.012	200	ANOVA test
Etiology			
Unknown	0.013	222	Area: 0.409
Alcohol	0.020	347	Intensity:0.459
HBV	0.007	111	ANOVA test
HCV	0.012	200	

Although statistically not significant, the mean value of both CD34 area and intensity are higher in HCC with lymphvascular space invasion, high histological grade, intermediate tumor size, and the underlying etiology of hepatitis C and alcoholic steatohepatitis.

## Abbreviations

CL: cirrhotic liver
HCC-CB: non-neoplastic cirrhotic background liver tissue adjacent to the tumor
HCC-C: tumor tissue of HCC with cirrhosis
HCC-NCB: non-neoplastic non-cirrhotic background liver tissue adjacent to the tumor
HCC-NC: tumor tissue of HCC without cirrhosis
MVD: microvessel density
NL: normal liver
